# Supplementary material for: Dynamic transcriptomic profiles of zebrafish gills in response to zinc supplementation
Source: BMC Genomics. 2010 Oct 11;11:553. doi: 10.1186/1471-2164-11-553 (PMC3091702; doi:10.1186/1471-2164-11-553)
Supplement: Additional file 2 — Interactive Direct Interaction Network representing the molecular interactions between zinc, copper, iron, calcium and proteins encoded by transcripts changed by zinc supplementation. Mini web-site containing index.html and hyperlinked pages in subdirectory describing a Direct Interaction Network automatically generated based on curated interactions contained within the proprietary PathwayArchitect database. Ovals represent proteins and the circles symbolize metal ions. Objects are coloured by their abundance in zebrafish at the time-point they were significantly different from the control is a scale from -4 fold (dark green) to +4 fold (dark red). Where significant differences were found at more than one time-point, the colour overlay shows expression at the first instance. Dark blue squares denote 'binding', and light blue squares 'expression'; green squares stand for 'regulation', green diamonds for 'metabolism', and green circles for 'promoter binding'. Arrow heads indicate directionality of the interaction where annotated. All nodes and edges can be further interrogated by selecting the relative area of the image. [file 1471-2164-11-553-S2.zip › PathwayArchitect Zn xs DIN/525187.html]

# REGULATION:

|  |  |
| --- | --- |
| Type | REGULATION |
| Effect | None |


---

|  |  |
| --- | --- |
| Score | 0 |


---

|  |  |
| --- | --- |
| Reference Count | 12 |


---

|  |  |
| --- | --- |
| Mechanism | Unknown |


---

|  |  |
| --- | --- |
| Reference:0 || Sentence | "OBJECTIVES: The association between the polymorphisms of three different antioxidative enzyme - catalase, copper-zinc superoxide dismutase (Cu/ Zn-SOD) and extracellular superoxide dismutase (EC-SOD) - genes and macroangiopathy was examined in patients with type 2 diabetes mellitus." |
| PMID | 11350569 |
| Year | 2001 |
| Species | Human |
| Journal | J Intern Med |
| RefScore | 1 |
| Source | PArchNLP |
  |
|  |  |


---

|  |  |
| --- | --- |
 Reference:1 || Sentence | "We conducted a case-control study to evaluate the association between serum levels of copper/zinc superoxide dismutase (Cu/Zn SOD) and the risk of gastric cancer." |
| PMID | 12417035 |
| Year | 2002 |
| Species | Human |
| Journal | Jpn J Cancer Res |
| RefScore | 0 |
| Source | PArchNLP |
  ||  |  |


---

|  |  |
| --- | --- |
 Reference:2 || Sentence | "We found that while transcriptional induction by zinc can be achieved by elevated zinc concentration alone, induction by cadmium, copper, or H2O2 additionally requires the presence of zinc-saturated metallothionein." |
| PMID | 14612393 |
| Year | 2003 |
| Species | Human |
| Journal | Mol Cell Biol |
| RefScore | 0 |
| Source | PArchNLP |
  ||  |  |


---

|  |  |
| --- | --- |
 Reference:3 || Sentence | The effects of zinc in increasing calcium content and alkaline phosphatase activity in the diaphyseal and metaphyseal tissues were significantly weakened in the presence of copper (10(-4) M). |
| Year | 2005 |
| PMID | 16327168 |
| Species | Rat |
| Journal | Biol Pharm Bull |
| RefScore | 2 |
| Source | PArchNLP |
  ||  |  |


---

|  |  |
| --- | --- |
 Reference:4 || Sentence | The objectives of this study were to examine the association between urinary excretion of cadmium (U-Cd), copper (U-Cu), and zinc (U-Zn) and the severity of two different indicators of renal toxicity (urinary excretion of beta2-microglobulin [U-beta2-MG] and metallothionein [U-MT]) in Cd-exposed subjects compared to controls, and to assess the physiologic mechanisms by which the exposure to environmental Cd affects U-Cd, U-Cu, and U-Zn. |
| Year | 2005 |
| PMID | 16327056 |
| Species | Human |
| Journal | Biol Trace Elem Res |
| RefScore | 1 |
| Source | PArchNLP |
  ||  |  |


---

|  |  |
| --- | --- |
 Reference:5 || Sentence | The recombinant SBP domain of Crr1 shows zinc-dependent binding to functionally defined copper-response elements associated with the CYC6 and CPX1 promoters that contain a critical GTAC core sequence. |
| Year | 2005 |
| PMID | 16352720 |
| Journal | Proc Natl Acad Sci U S A |
| RefScore | 1 |
| Source | PArchNLP |
  ||  |  |


---

|  |  |
| --- | --- |
 Reference:6 || Sentence | Oral zinc treatment can lead to symptomatic copper deficiency in susceptible patients. |
| Year | 2004 |
| PMID | 15333196 |
| Species | Human |
| Journal | Ann Clin Biochem |
| RefScore | 1 |
| Source | PArchNLP |
  ||  |  |


---

|  |  |
| --- | --- |
 Reference:7 || Sentence | Tissue zinc and copper levels did not depict any significant depletion, although changes like marked depletion of zinc following CaNa(2)EDTA and copper after MiADMSA administration were of some concern. |
| PMID | 15452883 |
| Year | 2004 |
| Species | Rat |
| Journal | J Biochem Mol Toxicol |
| RefScore | 3 |
| Source | PArchNLP |
  ||  |  |


---

|  |  |
| --- | --- |
 Reference:8 || Sentence | The aim of this study was to determine the effects of oral zinc treatment on red cell copper/zinc-superoxide dismutase (Cu/Zn-SOD) activity and zinc and copper concentrations in growth retardation. |
| PMID | 15621927 |
| Year | 2004 |
| Species | Human |
| Journal | Biol Trace Elem Res |
| RefScore | 1 |
| Source | PArchNLP |
  ||  |  |


---

|  |  |
| --- | --- |
 Reference:9 || Sentence | OBJECTIVE: To observe the effects of high level Zn intake on zinc (Zn) copper (Cu), lipids metabolism and antioxidation function in man so as to provide scientific basis for Zn supplementation. |
| PMID | 15727190 |
| Year | 2004 |
| Species | Human |
| Journal | Wei Sheng Yan Jiu |
| RefScore | 1 |
| Source | PArchNLP |
  ||  |  |


---

|  |  |
| --- | --- |
 Reference:10 || Sentence | OBJECTIVES: To evaluate the effects of zinc supplementation on serum zinc and copper levels, and the severity of adverse reactions and virologic responses in chronic hepatitis C patients undergoing interferon (IFN)/ribavirin therapy. |
| PMID | 15904908 |
| Year | 2005 |
| Species | Human |
| Journal | Clin Biochem |
| RefScore | 0 |
| Source | PArchNLP |
  ||  |  |


---

|  |  |
| --- | --- |
 Reference:11 || Sentence | Determinants of serum copper, zinc and selenium in healthy subjects. |
| PMID | 16168192 |
| Year | 2005 |
| Species | Human |
| Journal | Ann Clin Biochem |
| RefScore | 0 |
| Source | PArchNLP |
  |


---

|  |  |
| --- | --- |
